# Crystal structure of bis­{(3,5-di­methyl­pyrazol-1-yl)di­hydro­[3-(pyridin-2-yl)pyrazol-1-yl]­borato}iron(II)

**DOI:** 10.1107/S2056989020009214

**Published:** 2020-07-10

**Authors:** Sascha Ossinger, Christian Näther, Felix Tuczek

**Affiliations:** a Institut für Anorganische Chemie, Christian-Albrechts-Universität Kiel, Max-Eyth-Str. 2, D-24118 Kiel, Germany

**Keywords:** crystal structure, di­hydro­(pyrazole)(pyridlypyrazole)­borate derivative, Fe^II^, discrete complex.

## Abstract

The structure determination of [Fe(C_13_H_15_BN_5_)_2_] was undertaken as part of a project on the modification of the recently published spin-crossover (SCO) complex [Fe{H_2_B(pz)(pypz)}_2_] (pz = pyrazole, pypz = pyridyl­pyrazole). To this end, a new ligand was synthesized in which two additional methyl groups are present. Its reaction with iron tri­fluoro­methane­sulfonate led to a pure sample of the title compound, as proven by X-ray powder diffraction. The asymmetric unit consists of one complex mol­ecule in a general position. The Fe^II^ atom is coordinated by two tridentate N-binding {H_2_B(3,5-(CH_3_)_2_-pz)(pypz)}^−^ ligands. The Fe—N bond lengths range between 2.1222 (13) and 2.3255 (15) Å, compatible with Fe^II^ in the high-spin state, which was also confirmed by magnetic measurements. Other than a very weak C—H⋯N non-classical hydrogen bond linking individual mol­ecules into rows extending parallel to [010], there are no remarkable inter­molecular inter­actions.

## Abstract 2

The Fe^II^ atom in the title complex, [Fe{H_2_B(3,5-(CH_3_)_2_-pz)(pypz)}_2_] (pz = pyrazole, pypz = pyridyl­pyrazole), is coordinated by two tridentate {H_2_B(pz)(pypz)}^−^ ligands in form of a distorted N_6_ octa­hedron.

## Chemical context   

**Figure 1:**
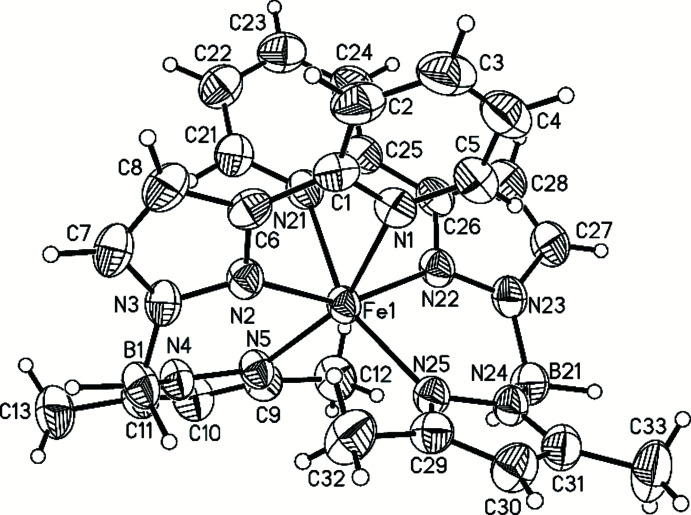
The mol­ecular structure of the title compound with the atom labelling and displacement ellipsoids drawn at the 50% probability level.

Spin-crossover (SCO) complexes of transition-metal cations (3*d* ^4^–3*d* ^7^) are a fascinating class of functional materials with potential for applications in electronic data storage or in spintronics (Gütlich *et al.*, 2013 ▸; Halcrow, 2013 ▸). The transition between the diamagnetic low-spin state (*S* = 0 for Fe^II^) and the paramagnetic high-spin state (*S* = 2 for Fe^II^) of such complexes can be induced *via* temperature or light as stimuli. In most cases, SCO complexes are based on octa­hedral [Fe^II^N_6_] coordination spheres with chelating or mono-coordinating nitro­gen donor ligands, because these combinations lead to the largest metal–ligand bond length differences between the two spin states and the largest lifetimes of the photoexcited spin states (Halcrow, 2007 ▸). Whereas hundreds of Fe^II^ SCO complexes have been reported (Halcrow, 2007 ▸), only a few of them are based on organoborate ligands such as [Fe(H_2_B(pz)_2_)_2_(*L*)] (pz = pyrazole; *L* = di-imine co-ligand) or tripodal organoborate ligands such as [Fe(HB(pz)_3_] (pz = pyrazole and derivatives thereof). These compounds are of special inter­est because most of them, as we and other research groups have shown, are suitable for physical vapour deposition, which is one important requirement for a possible application of these materials (Ruben & Kumar, 2019 ▸; Naggert *et al.*, 2015 ▸; Ossinger *et al.*, 2020*a* ▸). Notably, bidentate compounds of the type [Fe(H_2_B(pz)_2_)_2_(*L*)] have been found to dissociate into the tetra­hedral complex [Fe(H_2_B(pz)_2_)_2_] and the free co-ligand (Gopakumar *et al.*, 2013 ▸) in the first (sub)monolayer on Au(111), whereas the SCO complex [Fe(HB(3,5-(CH_3_)_2_-pz)_3_)_2_] supported by a tridentate tris(pyrazol­yl)borate ligand can be adsorbed without fragmentation on an Au(111) surface in a submonolayer (Bairagi *et al.*, 2016 ▸, 2018 ▸). Along these lines, we synthesized and characterized the first neutral and vacuum-evaporable SCO complex based on a linear tridentate organoborate ligand. The new complex [Fe{H_2_B(pz)(pypz)}_2_] was found to crystallize in two polymorphs, **I** (*T*_1/2_ = ∼270 K) and **II** (T_1/2_ = ∼390 K), with form **II** exhibiting π–π inter­actions that are absent in form **I** (Ossinger *et al.*, 2020*c* ▸). To investigate a possible correlation between the spin-transition temperature (*T*_1/2_) and the presence of π–π inter­actions in more detail, we decided to modify the complex [Fe{H_2_B(pz)(pypz)}_2_] by replacing 1*H*-pyrazole with 3,5-dimethyl-pyrazole in the tridentate ligand. This led to the title complex, [Fe{H_2_B(3,5-(CH_3_)_2_-pz)(pypz)}_2_], which was characterized by single crystal X-ray diffraction. The corresponding X-ray powder diffraction pattern revealed that the employed synthetic route yields a pure complex (see Fig. 1 ▸ in the supporting information). It was found to be suitable for physical vapour deposition, in analogy to the parent system [Fe{H_2_B(pz)(pypz)}_2_] (Ossinger *et al.*, 2020*c* ▸). Comparison of the infrared spectra from the bulk and the vacuum-deposited compound shows identical vibrational modes, indicating that no decomposition takes place upon vacuum evaporation and deposition (Fig. S2). Magnetic measurements revealed the presence of the high-spin state in the temperature range from 25 K to 300 K (Fig. S3), in contrast to the parent system and its two polymorphs, which exhibit the low-spin in polymorph **II** and SCO behaviour in polymorph **I**. Moreover, the crystal structure of the title compound is devoid of π–π inter­actions, similar to polymorph **I** of the parent complex [Fe{H_2_B(pz)(pypz)}_2_]. As the latter shows thermally induced spin crossover, this indicates that the introduction of methyl groups has shifted the magnetic properties of the parent complex into the high-spin regime.
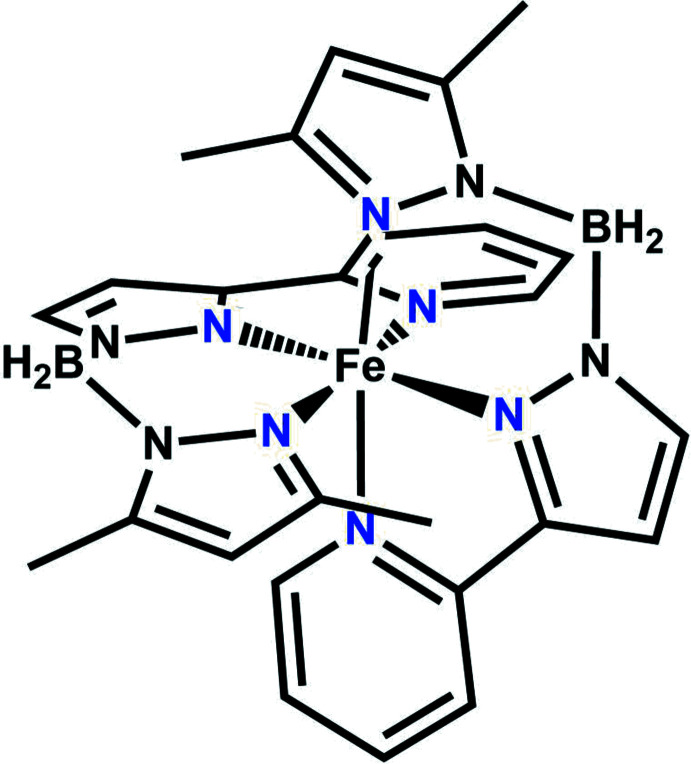


## Structural commentary   

The asymmetric unit of the title compound consists of one discrete complex in a general position. The central Fe^II^ atom is coordinated by six N atoms of two tridentate mono-anionic {H_2_B(3,5-(CH_3_)_2_-pz)(pypz)} ligands in a slightly distorted octa­hedral environment (Fig. 1 ▸), as shown by different bond lengths and angles deviating from ideal values (Table 1 ▸). The Fe—N bond lengths involving the N(pz) atoms are 2.1222 (13), 2.1264 (13), 2.1782 (14) and 2.1866 (14) Å and thus are significantly shorter than those to the N(py) atoms [2.2972 (14) and 2.3255 (15) Å]. The average bond length is 2.206 Å and thus in the range expected for Fe^II^ atoms in the high-spin state.

To characterize the distortion in more detail, the structural parameters Σ and Θ were calculated with the aid of the program *OctaDist* (OctaDist, 2019 ▸). Σ is calculated from the 12 *cis*-N—Fe—N angles and is a general measure of the deviation from an ideal octa­hedron. Θ is calculated from 24 unique N—Fe—N angles measured on the projection of two triangular faces of the octa­hedron along their common pseudo-threefold axis and indicates more specifically its distortion from an octa­hedral towards a trigonal–prismatic structure. For a perfectly octa­hedral complex Σ = Θ = 0 is valid (Guionneau *et al.*, 2004 ▸; Iasco *et al.*, 2017 ▸; Halcrow, 2013 ▸).

For the title compound, the values Σ =119.92° and Θ = 337.22° were calculated, which are significantly higher than those in the polymorphic modifications **I** (Σ = 92.12°, Θ = 298.06°) and **II** (Σ = 47.43°, Θ = 149.08°) of the parent system (Ossinger *et al.*, 2020*c* ▸ **)**.

## Supra­molecular features   

**Figure 2:**
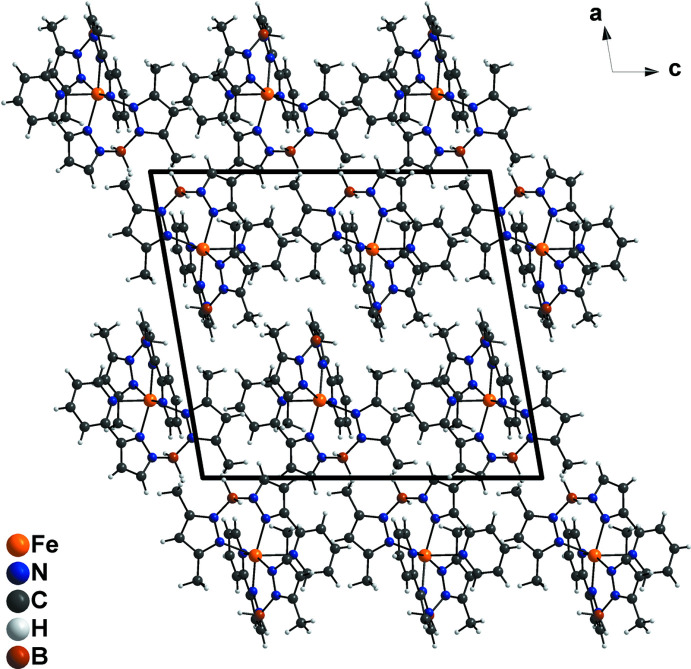
Crystal structure of the title compound in a view along [010].

In polymorph **II** of the parent system [Fe{H_2_B(pz)(pypz)}_2_], individual complexes are pairwise linked to dimers by inter­molecular π–π inter­actions between the pyridine rings of the ligands of neighbouring complexes (Ossinger *et al.*, 2020*c* ▸). In the crystal structure of the title compound, no parallel arrangements of pyridine rings and no inter­molecular π–π inter­actions are observed (Fig. 2 ▸), as was the case for polymorph **I** of [Fe{H_2_B(pz)(pypz)}_2_].

Apart from a weak C—H⋯N hydrogen bond (Table 2 ▸) that links neighbouring mol­ecules into rows extending parallel to [010], there are no remarkable inter­molecular inter­actions other than van der Waals forces.

## Database survey   

There are at least 21 crystal structures of iron complexes with di­hydro-bis­(pyrazol-1-yl)borate and different co-ligands reported in the literature, which include, for example, [Fe(H_2_B(pz)_2_)_2_(phen)] and [Fe(H_2_B(pz)_2_)_2_(2,2′-bipy)] (Real *et al.*, 1997 ▸; Thompson *et al.*, 2004 ▸) as the most well-known complexes. In the others, the co-ligand is exchanged by annelated bipyridyl ligands (Kulmaczewski *et al.*, 2014 ▸), various modified di­aryl­ethene ligands (Nihei *et al.*, 2013 ▸; Milek *et al.*, 2013 ▸; Mörtel *et al.*, 2017 ▸, 2020 ▸), 4,7-dimethyl-phenanthroline (Naggert *et al.*, 2015 ▸), di­methyl­bipyridine derivatives substituted in the 5,5′ position (Xue *et al.*, 2018 ▸), di­amino­bipyridine (Luo *et al.*, 2016 ▸), chiral (*R*)/(*S*)-4,5-pinenepyridyl-2-pyrazine ligands (Ru *et al.*, 2017 ▸) and further ligands with methyl substituents at the pyrazole unit or co-ligand unit, which also includes different solvates (Ossinger *et al.*, 2019 ▸, 2020*a* ▸,*b* ▸). In all of these complexes, the Fe^II^ atoms are coordinated by three bidentate chelate ligands in a distorted octa­hedral environment, and spin-crossover behaviour is observed. Moreover, the crystal structure of the synthetic inter­mediate ([Fe(H_2_B(pz)_2_)_2_(MeOH)_2_]) used for the preparation of the Fe–phenanthroline complex has also been published (Ossinger *et al.*, 2016 ▸).

Furthermore, numerous crystal structures of iron complexes based on the tripodal hydro­tris­(pyrazol-1-yl)borate ligand with different modifications of the pyrazole unit (Oliver *et al.*, 1980 ▸; Calogero *et al.*, 1994 ▸; Rheingold *et al.*, 1997 ▸; Cecchi *et al.*, 2001 ▸; Reger *et al.*, 2006 ▸; Ni *et al.*, 2011 ▸; Salmon *et al.*, 2009 ▸) and/or another fourth substituent in place of the hydrogen atom (Sohrin *et al.*, 1995 ▸; Reger *et al.*, 2005*a* ▸,*b* ▸) or triazole (Janiak, 1994 ▸) have been reported in the literature.

## Synthesis and crystallization   

All reactions were carried out in dry solvents, and the complexation was carried out under nitro­gen-atmosphere using standard Schlenk techniques or in an M-Braun Labmaster 130 glovebox under argon.

3,5-Di­methyl­pyrazole, 2-(1*H*-pyrazol-3-yl)pyridine and potassium tetra­hydro­borate were purchased from commercial sources and were used without further purification. Iron(II) triflate, which is also commercially available, was purified by the following method: The compound was dissolved in dry methanol (a few ml for a supersaturated solution), filtered off and afterwards the solvent was removed *in vacuo*. Solvents were purchased from commercial sources and purified by distillation over conventional drying agents.

**Synthesis of K[H_2_B(3,5-(CH_3_)_2_-pz)(pypz)]:** Potassium tetra­hydro­borate (539 mg, 0.01 mol), 3,5-di­methyl­pyrazole (961 mg, 0.01 mol) and 2-(1*H*-pyrazol-3-yl)pyridine (1.45 g, 0.01 mol) were suspended in toluene (20 ml) and refluxed for 17 h. The solution was filtered whilst hot to remove any residual traces of unreacted K[BH_4_]. The filtrate was allowed to cool to room temperature. A few hours later a white precipitate formed, and after one additional night of crystallization the precipitate was collected by suction filtration and subsequently dissolved in a few ml of aceto­nitrile. The resulting cloudy solution was again filtered by suction filtration. The solvent was removed *in vacuo*, and a white precipitate was obtained. Yield 260 mg (859 µmol, 9% *vs* K[BH_4_]).

**Elemental analysis** calculated for C_13_H_15_BKN_5_: C 53.62, H 5.19, N 24.05%, found: C 53.63, H 4.99, N 23.75%.

**HRESI–MS(+)(CH_3_CN):** *m*/*z* (%) = [*M* − K + 2H]^+^ calc­ulated 254.15715, found 254.15683 (100).

**^1^H NMR (500 MHz, CD_3_CN):** δ/ppm = 8.49 (*ddd*, *J* = 4.9 Hz, 1.8 Hz, 1.0 Hz, 1H, py-H^8^), 7.72 (*ddd*, *J* = 8.0 Hz, 1.5 Hz, 1.0 Hz, 1H, py-H^10^), 7.68 (*ddd*, *J* = 8.0 Hz, 1.5 Hz, 1.0 Hz, 1H, py-H^7^), 7.50 (*d*, *J* = 2.0 Hz, 1H, pz-H^5^), 7.14 (*ddd*, *J* = 7.2 Hz, 4.9 Hz, 1.5 Hz, 1H, py-H^9^), 6.56 (*d*, *J* = 2.0 Hz, 1H, pz-H^4^), 5.58 (*s*, 1H, pz-H^4A^), 3.49 (*dd*, *J* = 187.8 Hz, 69.1 Hz, 2H, B-H), 2.24 (*d*, *J* = 0.6 Hz, 3H, pz-Me), 2.10 [*m*(*d*), *J* = 0.5 Hz, 3H, pz-Me].

**^13^C{^1^H} NMR (125 MHz, CD_3_CN):** δ/ppm = 154.72 (C_q_, py-C^6^), 151.82 (C_q_, pz-C^3^), 150.20 (CH, py-C^8^), 146.79 (C_q_, pz-C^3A^ or C^5A^), 143.83 (C_q_, pz-C^3A^ or C^5A^), 137.41 (CH, py-C^7^), 136.31 (CH, pz-C^5^), 122.18 (CH, py-C^9^), 120.53 (CH, py-C^10^), 104.21 (CH, pz-C^4A^), 102.86 (CH, pz-C^4^), 13.63 (CH_3_, pz-Me), 13.04 (CH_3_, pz-Me).

**^11^B NMR (160 MHz, CD_3_CN):** δ/ppm = −9.32 (*t*, *J* = 98.6 Hz, 1B).

**IR (ATR):** ν/cm^−1^ = 3069, 3048, 3022, 3005 (*w*, ν[=C—H]), 2952, 2917, 2907, 2860, 2815 (*w*, ν[–CH_3_]), 2362, 2325 (*m*, ν_asym._[–BH_2_]), 2264, 2250 (*m*, ν_sym._[–BH_2_]), 1695 (*w*), 1592 (*s*), 1566 (*m*), 1533 (*m*), 1511 (*m*), 1486 (*m*), 1425 (*s*), 1378 (*w*), 1352 (*m*), 1276 (*w*), 1225 (*m*), 1180 (*s*), 1158 (*s*), 1145 (*s*), 1125 (*s*), 1086 (*s*), 1056 (*s*), 1029 (*m*), 994 (*m*), 980 (*m*), 955 (*m*), 896 (*m*), 849 (*m*), 792 (*w*), 780 (*m*), 747 (*s*), 721 (*m*), 706 (*m*), 688 (*m*), 672 (*w*), 653 (*w*), 643 (*m*), 506 (*w*), 459 (*w*), 400 (*m*).

**Raman (Bulk):** ν/cm^−1^ = 3134, 3119, 3069, 3055, 3010 (*w*, ν[=C—H]), 2972, 2953, 2923, 2865 (*w*, ν[–CH_3_]), 2472, 2382, 2365, 2333 (*vw*, ν_asym._[–BH_2_]), 2267 (*vw*, ν_sym._[–BH_2_]), 1594 (*s*), 1567 (*w*), 1513 (*s*), 1490 (*w*), 1442 (*w*), 1358 (*m*), 1279 (*w*), 1261 (*w*), 1237 (*w*), 1226 (*w*), 1184 (*w*), 1148 (*w*), 1129 (*w*), 1090 (*w*), 1049 (*w*), 1031 (*w*), 993 (*m*), 960 (*m*), 796 (*w*), 781 (*w*), 707 (*w*), 621 (*w*), 589 (*w*).

**Synthesis of [Fe{H_2_B(3,5-(CH_3_)_2_-pz)(pypz)}_2_]:** To a solution of Fe(OTf)_2_ (124 mg, 351 µmol) in methanol (1 ml) a solution of K[H_2_B(3,5-(CH_3_)_2_-pz)(pypz)] (203 mg, 698 µmol) in methanol (4 ml) was added dropwise, leading to the formation of a dark-yellow-coloured solution. Immediately, a dark-yellow-coloured precipitate was formed. The suspension was stirred for 15 min at room temperature, and then the precip­itate was filtered off, washed with methanol (5 ml) and dried under reduced pressure (1 h). Yield: 128 mg (229 µmol, 65% vs. Fe(OTf)_2_).

**Elemental analysis** calculated for C_26_H_30_B_2_FeN_10_: C 55.76, H 5.4, N 25.01%, found: C 55.92, H 5.26, N 24.79%.

**HRESI–MS(+)(CH_3_CN+MeOH):** *m*/*z* (%) = [*M* + H]^+^ calc­ulated 561.22632, found 561.22575 (100).

**IR (ATR):** ν/cm^−1^ = 3138, 3118, 3079, 3060 (*w*, ν[=C—H]), 2979, 2960, 2924, 2858 (*w*, ν[–CH_3_]), 2417, 2364, 2303 (*m*, ν_asym._[–BH_2_]), 2266 (*w*, ν_sym._[–BH_2_]), 1605 (*m*), 1566 (*w*), 1537 (*m*), 1488 (*w*), 1445 (*w*), 1433 (*m*), 1421 (*m*), 1376 (*m*), 1354 (*m*), 1294 (*w*), 1249 (*w*), 1196 (*m*), 1170 (*s*), 1156 (*m*), 1102 (*m*), 1094 (*m*), 1072 (*s*), 1041 (*m*), 1017 (*w*), 982 (*w*), 962 (*w*), 880 (*m*), 862 (*w*), 792 (*w*), 764 (*s*), 723 (*m*), 706 (*w*), 686 (*m*), 670 (*w*), 655 (*m*), 635 (*m*), 608 (*w*), 510 (*w*), 482 (*m*), 459 (*m*), 410 (*m*).

**Raman (Bulk):** ν/cm^−1^ = 3140, 3061 (*w*, ν[=C—H]), 2931 (*m*, ν[–CH_3_]), 2330 (*vw*, ν_asym._[–BH_2_]), 2274 (*vw*, ν_sym._[–BH_2_]), 1653 (*w*), 1606 (*s*), 1566 (*m*), 1527 (*s*), 1489 (*m*), 1444 (*w*), 1356 (*s*), 1007 (*m*), 966 (*w*).

**UV/Vis (KBr):** λ_*max*_/nm = 204, 253, 300, 392–552 (*br*).

**Crystallization:** Single crystals of the compound were obtained under a nitro­gen atmosphere by resolving microcrystalline material in dry toluene that was overlayed with dry *n*-hexane. This mixture was stored at 278 K, and after a few weeks long orange-coloured needle-like single crystals were formed.

**Experimental details:** NMR spectra were recorded in deuterated solvents on a Bruker DRX500 spectrometer operating at a ^1^H frequency of 500 MHz, a ^13^C frequency of 125 MHz, and a ^11^B frequency of 160 MHz. They were referenced to the residual protonated solvent signal [^1^H: δ(CD_3_CN) = 1.94 ppm], the solvent signal [^13^C: δ(CD_3_CN) = 118.26 ppm], or an external standard (^11^B: BF_3_·Et_2_O) (Gottlieb *et al.*, 1997 ▸; Fulmer *et al.*, 2010 ▸). Signals were assigned with the help of DEPT-135 and two-dimensional correlation spectra (^1^H,^1^H-COSY, ^1^H,^13^C-HSQC, and ^1^H,^13^C-HMBC). Signal multiplicities are abbreviated as *s* (singlet), *d* (doublet), *t* (triplet), *m* (multiplet), and *br* (broad signal). Elemental analyses were performed using a vario MICRO cube CHNS element analyser from Elementar. Samples were burned in sealed tin containers by a stream of oxygen. High-resolution ESI mass spectra were recorded on a ThermoFisher Orbitrap spectrometer. IR spectra were recorded on a Bruker Alpha-P ATR–IR Spectrometer. Signal intensities are marked as *s* (strong), *m* (medium), *w* (weak) and *br* (broad). For FT–Raman spectroscopy, a Bruker RAM II-1064 FT-Raman Module, a R510-N/R Nd:YAG-laser (1046 nm, up to 500 mW) and a D418-T/R liquid-nitro­gen-cooled, highly sensitive Ge detector or a Bruker IFS 66 with a FRA 106 unit and a 35mW Nd:YAG-laser (1064 nm) were used. XRPD experiments were performed with a Stoe Transmission Powder Diffraction System (STADI P) with Cu *K*α radiation (λ = 1.5406 Å) equipped with a position-sensitive detector (Mythen-K1). UV/vis spectra were recorded with a Cary 5000 spectrometer in transmission geometry. The magnetic measurement was performed at 1 T between 300 and 2 K using a physical property measurement system (PPMS) from Quantum Design. Diamagnetic corrections were applied with the use of Pascal’s constants (Bain & Berry, 2008 ▸).

## Refinement   

Crystal data, data collection and structure refinement details are summarized in Table 3 ▸. C-bound hydrogen atoms were positioned with idealized geometry (methyl H atoms allowed to rotate but not to tip) and were refined with *U*_iso_(H) = 1.2*U*_eq_(C) (1.5 for methyl H atoms) using a riding model. B-bound hydrogen atoms were located in a difference-Fourier map and were refined freely.

## Supplementary Material

Crystal structure: contains datablock(s) I. DOI: 10.1107/S2056989020009214/wm5574sup1.cif

Structure factors: contains datablock(s) I. DOI: 10.1107/S2056989020009214/wm5574Isup2.hkl

Figure S1. Experimental XRPD pattern of [Fe{H2B(3,5-(CH3)2-pz)(pypz)}2] measured at rt (a) as well as simulated XRPD pattern calculated from the single crystal structure measured at 200 K (b). DOI: 10.1107/S2056989020009214/wm5574sup3.png

Figure S2. Fourier transform infrared (FT-IR) spectra of bulk material (black dashed line) and vacuum-deposited material (red line) of the title compound measured at rt. DOI: 10.1107/S2056989020009214/wm5574sup4.png

Figure S3. ChiT vs T curve of the title compound between 2 and 300 K. The curves resulting from cooling and heating the sample almost coincidence. DOI: 10.1107/S2056989020009214/wm5574sup5.png

CCDC reference: 2014411

Additional supporting information:  crystallographic information; 3D view; checkCIF report

## Figures and Tables

**Table 1:** Selected geometric parameters (Å, °)

Fe1—N2	2.1222 (13)	Fe1—N25	2.1866 (14)
Fe1—N22	2.1264 (13)	Fe1—N1	2.2972 (14)
Fe1—N5	2.1782 (14)	Fe1—N21	2.3255 (15)
			
N2—Fe1—N5	86.86 (5)	N5—Fe1—N1	157.65 (5)
N22—Fe1—N5	110.14 (5)	N25—Fe1—N1	87.65 (5)
N2—Fe1—N25	110.34 (5)	N2—Fe1—N21	86.01 (5)
N22—Fe1—N25	86.68 (5)	N22—Fe1—N21	72.10 (5)
N5—Fe1—N25	107.84 (5)	N5—Fe1—N21	89.10 (5)
N2—Fe1—N1	72.40 (5)	N25—Fe1—N21	156.60 (5)
N22—Fe1—N1	86.20 (5)	N1—Fe1—N21	81.38 (5)

**Table 2:** Hydrogen-bond geometry (Å, °)

*D*—H⋯*A*	*D*—H	H⋯*A*	*D*⋯*A*	*D*—H⋯*A*
C3—H3⋯N25^i^	0.95	2.60	3.502 (4)	159

Symmetry code: (i) 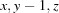.

**Table 3:** Experimental details

Crystal data	
Chemical formula	[Fe(C_13_H_15_BN_5_)_2_]
*M*_r_	560.07
Crystal system, space group	Monoclinic, *P*2_1_/*c*
Temperature (K)	200
*a*, *b*, *c* (Å)	17.1798 (6), 8.7991 (2), 18.7608 (7)
β (°)	99.711 (3)
*V* (Å^3^)	2795.37 (16)
*Z*	4
Radiation type	Mo *K*α
μ (mm^−1^)	0.58
Crystal size (mm)	0.20 × 0.12 × 0.08

Data collection	
Diffractometer	Stoe IPDS1
Absorption correction	Numerical (*X-RED* and *X-SHAPE*; Stoe & Cie, 2008 ▸)
*T*_min_, *T*_max_	0.805, 0.960
No. of measured, independent and observed [*I* > 2σ(*I*)] reflections	16844, 6071, 5067
*R*_int_	0.029
(sin θ/λ)_max_ (Å^−1^)	0.639

Refinement	
*R*[*F* ^2^ > 2σ(*F* ^2^)], *wR*(*F* ^2^), *S*	0.034, 0.091, 1.04
No. of reflections	6071
No. of parameters	372
H-atom treatment	H atoms treated by a mixture of independent and constrained refinement
Δρ_max_, Δρ_min_ (e Å^−3^)	0.24, −0.32

Computer programs: *X-AREA* (Stoe & Cie, 2008 ▸), *SHELXT* (Sheldrick, 2015*a* ▸), *SHELXL2018/3* (Sheldrick, 2015*b* ▸), *DIAMOND* (Brandenburg, 1999 ▸) and *publCIF* (Westrip, 2010 ▸).

## supplementary crystallographic information

### Crystal data

**Table:**

[Fe(C_13_H_15_BN_5_)_2_]	*F*(000) = 1168
*M**_r_* = 560.07	*D*_x_ = 1.331 Mg m^−^^3^
Monoclinic, *P*2_1_/*c*	Mo *K*α radiation, λ = 0.71073 Å
*a* = 17.1798 (6) Å	Cell parameters from 16844 reflections
*b* = 8.7991 (2) Å	θ = 2.2–27.0°
*c* = 18.7608 (7) Å	µ = 0.57 mm^−^^1^
β = 99.711 (3)°	*T* = 200 K
*V* = 2795.37 (16) Å^3^	Bar, orange
*Z* = 4	0.20 × 0.12 × 0.08 mm

### Data collection

**Table:**

Stoe IPDS-1 diffractometer	5067 reflections with *I* > 2σ(*I*)
ω scans	*R*_int_ = 0.029
Absorption correction: numerical (X-Red and X-Shape; Stoe & Cie, 2008)	θ_max_ = 27.0°, θ_min_ = 2.2°
*T*_min_ = 0.805, *T*_max_ = 0.960	*h* = −21→20
16844 measured reflections	*k* = −11→10
6071 independent reflections	*l* = −23→23

### Refinement

**Table:**

Refinement on *F*^2^	0 restraints
Least-squares matrix: full	Hydrogen site location: mixed
*R*[*F*^2^ > 2σ(*F*^2^)] = 0.034	H atoms treated by a mixture of independent and constrained refinement
*wR*(*F*^2^) = 0.091	*w* = 1/[σ^2^(*F*_o_^2^) + (0.0488*P*)^2^ + 0.5301*P*] where *P* = (*F*_o_^2^ + 2*F*_c_^2^)/3
*S* = 1.04	(Δ/σ)_max_ = 0.001
6071 reflections	Δρ_max_ = 0.24 e Å^−^^3^
372 parameters	Δρ_min_ = −0.32 e Å^−^^3^

### Special details

**Table:**

Geometry. All esds (except the esd in the dihedral angle between two l.s. planes) are estimated using the full covariance matrix. The cell esds are taken into account individually in the estimation of esds in distances, angles and torsion angles; correlations between esds in cell parameters are only used when they are defined by crystal symmetry. An approximate (isotropic) treatment of cell esds is used for estimating esds involving l.s. planes.

### Fractional atomic coordinates and isotropic or equivalent isotropic displacement parameters (Å^2^)

**Table:**

	*x*	*y*	*z*	*U*_iso_*/*U*_eq_	
Fe1	0.25357 (2)	0.66865 (3)	0.38412 (2)	0.03635 (8)	
N1	0.24645 (8)	0.42005 (16)	0.41986 (8)	0.0424 (3)	
C1	0.31541 (11)	0.34392 (19)	0.42876 (9)	0.0431 (4)	
C2	0.32056 (14)	0.1918 (2)	0.44860 (11)	0.0556 (5)	
H2	0.369711	0.139860	0.454262	0.067*	
C3	0.25320 (15)	0.1174 (2)	0.45993 (11)	0.0642 (6)	
H3	0.255326	0.013376	0.473603	0.077*	
C4	0.18277 (14)	0.1952 (2)	0.45128 (11)	0.0609 (5)	
H4	0.135633	0.145907	0.458790	0.073*	
C5	0.18194 (12)	0.3461 (2)	0.43149 (11)	0.0522 (4)	
H5	0.133329	0.399861	0.425881	0.063*	
C6	0.38277 (10)	0.43509 (19)	0.41776 (9)	0.0419 (4)	
N2	0.37079 (7)	0.58498 (16)	0.41015 (7)	0.0388 (3)	
N3	0.43977 (8)	0.64900 (17)	0.40183 (8)	0.0432 (3)	
C7	0.49500 (11)	0.5398 (2)	0.40489 (11)	0.0542 (5)	
H7	0.548765	0.555785	0.400833	0.065*	
C8	0.46140 (11)	0.4016 (2)	0.41481 (11)	0.0538 (5)	
H8	0.486204	0.304748	0.418770	0.065*	
B1	0.44835 (11)	0.8240 (3)	0.40255 (12)	0.0481 (5)	
H1A	0.5065 (13)	0.849 (2)	0.3886 (11)	0.059 (6)*	
H2A	0.4405 (12)	0.867 (2)	0.4583 (12)	0.057 (6)*	
N4	0.38384 (8)	0.89317 (17)	0.34347 (8)	0.0415 (3)	
N5	0.30409 (8)	0.87040 (16)	0.34245 (7)	0.0397 (3)	
C9	0.26662 (10)	0.9666 (2)	0.29304 (10)	0.0458 (4)	
C10	0.32095 (12)	1.0504 (2)	0.26233 (11)	0.0556 (5)	
H10	0.309803	1.125859	0.225828	0.067*	
C11	0.39404 (11)	1.0018 (2)	0.29549 (10)	0.0498 (4)	
C12	0.17874 (11)	0.9804 (2)	0.27768 (12)	0.0568 (5)	
H12A	0.157151	0.907477	0.240004	0.085*	
H12B	0.164113	1.083753	0.261118	0.085*	
H12C	0.157288	0.959006	0.321832	0.085*	
C13	0.47337 (13)	1.0549 (3)	0.28292 (14)	0.0679 (6)	
H13A	0.503941	1.091565	0.328528	0.102*	
H13B	0.466683	1.137548	0.247405	0.102*	
H13C	0.501442	0.970313	0.264603	0.102*	
N21	0.25062 (8)	0.55241 (17)	0.27238 (8)	0.0427 (3)	
C21	0.31204 (11)	0.5194 (2)	0.23987 (10)	0.0490 (4)	
H21	0.362874	0.554639	0.261370	0.059*	
C22	0.30524 (13)	0.4369 (2)	0.17666 (10)	0.0565 (5)	
H22	0.350227	0.416218	0.154950	0.068*	
C23	0.23111 (14)	0.3848 (3)	0.14559 (11)	0.0604 (5)	
H23	0.224711	0.325513	0.102665	0.072*	
C24	0.16692 (12)	0.4197 (2)	0.17750 (10)	0.0552 (5)	
H24	0.115568	0.386001	0.156645	0.066*	
C25	0.17838 (10)	0.5048 (2)	0.24059 (9)	0.0446 (4)	
C26	0.11446 (10)	0.5525 (2)	0.27746 (9)	0.0453 (4)	
N22	0.13333 (8)	0.64402 (16)	0.33500 (8)	0.0412 (3)	
N23	0.06650 (8)	0.67339 (18)	0.36111 (8)	0.0454 (3)	
C27	0.00588 (10)	0.6020 (3)	0.31960 (12)	0.0587 (5)	
H27	−0.047595	0.605253	0.326591	0.070*	
C28	0.03319 (11)	0.5240 (3)	0.26587 (12)	0.0602 (5)	
H28	0.003562	0.463864	0.228849	0.072*	
B21	0.06608 (11)	0.7902 (3)	0.42263 (12)	0.0480 (5)	
H21A	0.0796 (12)	0.901 (2)	0.4020 (11)	0.053 (5)*	
H21B	0.0062 (12)	0.785 (2)	0.4387 (10)	0.051 (5)*	
N24	0.13042 (7)	0.74699 (16)	0.48779 (8)	0.0406 (3)	
N25	0.20937 (7)	0.73351 (16)	0.48249 (7)	0.0385 (3)	
C29	0.24801 (10)	0.7151 (2)	0.55021 (9)	0.0433 (4)	
C30	0.19569 (11)	0.7175 (2)	0.59869 (10)	0.0527 (4)	
H30	0.208023	0.707301	0.649760	0.063*	
C31	0.12210 (11)	0.7378 (2)	0.55775 (10)	0.0503 (4)	
C32	0.33549 (11)	0.6980 (3)	0.56723 (11)	0.0631 (6)	
H32A	0.350805	0.600726	0.548199	0.095*	
H32B	0.352579	0.700541	0.619763	0.095*	
H32C	0.360596	0.781333	0.544921	0.095*	
C33	0.04415 (13)	0.7534 (4)	0.58257 (13)	0.0757 (7)	
H33A	0.021738	0.853680	0.568847	0.114*	
H33B	0.051594	0.742262	0.635260	0.114*	
H33C	0.008067	0.674317	0.559854	0.114*	

### Atomic displacement parameters (Å^2^)

**Table:**

	*U*^11^	*U*^22^	*U*^33^	*U*^12^	*U*^13^	*U*^23^
Fe1	0.02667 (11)	0.03770 (13)	0.04343 (13)	0.00015 (9)	0.00229 (8)	0.00072 (9)
N1	0.0431 (7)	0.0389 (7)	0.0438 (7)	−0.0020 (6)	0.0036 (6)	0.0004 (6)
C1	0.0525 (9)	0.0378 (8)	0.0387 (8)	0.0031 (7)	0.0069 (7)	−0.0024 (6)
C2	0.0756 (13)	0.0385 (10)	0.0546 (11)	0.0088 (9)	0.0164 (10)	0.0008 (8)
C3	0.1000 (17)	0.0387 (10)	0.0559 (11)	−0.0080 (11)	0.0187 (11)	0.0003 (8)
C4	0.0751 (14)	0.0506 (11)	0.0568 (11)	−0.0189 (10)	0.0104 (10)	0.0009 (9)
C5	0.0514 (10)	0.0516 (11)	0.0525 (10)	−0.0101 (8)	0.0052 (8)	0.0025 (8)
C6	0.0437 (8)	0.0409 (9)	0.0403 (8)	0.0105 (7)	0.0050 (6)	0.0008 (7)
N2	0.0298 (6)	0.0408 (7)	0.0446 (7)	0.0034 (5)	0.0028 (5)	0.0020 (6)
N3	0.0285 (6)	0.0516 (8)	0.0485 (8)	0.0053 (6)	0.0034 (5)	0.0039 (6)
C7	0.0349 (8)	0.0649 (12)	0.0631 (11)	0.0139 (8)	0.0089 (8)	0.0066 (9)
C8	0.0476 (10)	0.0521 (11)	0.0623 (11)	0.0191 (9)	0.0112 (8)	0.0038 (9)
B1	0.0322 (9)	0.0524 (11)	0.0569 (12)	−0.0038 (8)	−0.0007 (8)	0.0055 (9)
N4	0.0324 (6)	0.0429 (7)	0.0487 (8)	−0.0025 (6)	0.0056 (5)	0.0040 (6)
N5	0.0321 (6)	0.0399 (7)	0.0457 (7)	−0.0008 (5)	0.0029 (5)	0.0042 (6)
C9	0.0424 (9)	0.0439 (9)	0.0484 (9)	0.0041 (7)	0.0001 (7)	0.0061 (7)
C10	0.0565 (11)	0.0551 (11)	0.0542 (11)	0.0009 (9)	0.0063 (8)	0.0172 (9)
C11	0.0480 (10)	0.0496 (10)	0.0531 (10)	−0.0051 (8)	0.0119 (8)	0.0061 (8)
C12	0.0439 (10)	0.0586 (12)	0.0633 (11)	0.0090 (8)	−0.0037 (8)	0.0111 (9)
C13	0.0558 (12)	0.0706 (14)	0.0809 (15)	−0.0096 (11)	0.0219 (11)	0.0160 (12)
N21	0.0387 (7)	0.0448 (8)	0.0434 (7)	−0.0010 (6)	0.0035 (6)	0.0019 (6)
C21	0.0448 (9)	0.0538 (11)	0.0487 (10)	0.0018 (8)	0.0084 (7)	0.0032 (8)
C22	0.0631 (12)	0.0607 (12)	0.0475 (10)	0.0071 (10)	0.0143 (9)	0.0027 (9)
C23	0.0767 (14)	0.0591 (12)	0.0445 (10)	0.0002 (11)	0.0076 (9)	−0.0054 (9)
C24	0.0570 (11)	0.0583 (12)	0.0469 (10)	−0.0084 (9)	−0.0009 (8)	−0.0045 (8)
C25	0.0430 (9)	0.0455 (9)	0.0429 (8)	−0.0034 (7)	0.0005 (7)	0.0013 (7)
C26	0.0375 (8)	0.0502 (10)	0.0448 (9)	−0.0061 (7)	−0.0030 (7)	−0.0013 (7)
N22	0.0298 (6)	0.0448 (8)	0.0469 (7)	−0.0006 (5)	0.0005 (5)	−0.0006 (6)
N23	0.0272 (6)	0.0539 (9)	0.0531 (8)	−0.0012 (6)	0.0013 (5)	−0.0010 (7)
C27	0.0305 (8)	0.0751 (13)	0.0670 (12)	−0.0082 (9)	−0.0016 (8)	−0.0109 (10)
C28	0.0399 (9)	0.0740 (14)	0.0623 (12)	−0.0125 (9)	−0.0039 (8)	−0.0153 (10)
B21	0.0317 (9)	0.0549 (12)	0.0556 (11)	0.0055 (8)	0.0020 (8)	−0.0017 (9)
N24	0.0300 (6)	0.0438 (8)	0.0483 (8)	−0.0002 (6)	0.0072 (5)	−0.0011 (6)
N25	0.0297 (6)	0.0402 (7)	0.0449 (7)	0.0023 (5)	0.0042 (5)	−0.0005 (6)
C29	0.0397 (8)	0.0431 (9)	0.0455 (9)	0.0056 (7)	0.0022 (7)	−0.0012 (7)
C30	0.0512 (10)	0.0627 (11)	0.0440 (9)	0.0047 (9)	0.0073 (8)	0.0016 (8)
C31	0.0426 (9)	0.0576 (11)	0.0524 (10)	−0.0021 (8)	0.0125 (7)	−0.0007 (8)
C32	0.0428 (10)	0.0910 (16)	0.0515 (10)	0.0188 (10)	−0.0031 (8)	−0.0079 (10)
C33	0.0482 (11)	0.118 (2)	0.0653 (13)	−0.0035 (13)	0.0219 (10)	−0.0001 (14)

### Geometric parameters (Å, º)

**Table:**

Fe1—N2	2.1222 (13)	C9—C12	1.493 (2)
Fe1—N22	2.1264 (13)	C10—C11	1.372 (3)
Fe1—N5	2.1782 (14)	C11—C13	1.497 (3)
Fe1—N25	2.1866 (14)	N21—C21	1.337 (2)
Fe1—N1	2.2972 (14)	N21—C25	1.350 (2)
Fe1—N21	2.3255 (15)	C21—C22	1.379 (3)
N1—C5	1.334 (2)	C22—C23	1.386 (3)
N1—C1	1.347 (2)	C23—C24	1.375 (3)
C1—C2	1.388 (2)	C24—C25	1.386 (3)
C1—C6	1.451 (3)	C25—C26	1.455 (3)
C2—C3	1.377 (3)	C26—N22	1.342 (2)
C3—C4	1.376 (3)	C26—C28	1.399 (2)
C4—C5	1.378 (3)	N22—N23	1.347 (2)
C6—N2	1.339 (2)	N23—C27	1.346 (2)
C6—C8	1.393 (2)	N23—B21	1.547 (3)
N2—N3	1.3449 (19)	C27—C28	1.366 (3)
N3—C7	1.345 (2)	B21—N24	1.551 (2)
N3—B1	1.547 (3)	N24—C31	1.346 (2)
C7—C8	1.372 (3)	N24—N25	1.3816 (18)
B1—N4	1.553 (2)	N25—C29	1.340 (2)
N4—C11	1.345 (2)	C29—C30	1.382 (3)
N4—N5	1.3817 (18)	C29—C32	1.490 (2)
N5—C9	1.338 (2)	C30—C31	1.376 (3)
C9—C10	1.388 (3)	C31—C33	1.496 (3)
			
N2—Fe1—N22	151.54 (6)	N5—C9—C10	110.18 (15)
N2—Fe1—N5	86.86 (5)	N5—C9—C12	122.54 (17)
N22—Fe1—N5	110.14 (5)	C10—C9—C12	127.25 (17)
N2—Fe1—N25	110.34 (5)	C11—C10—C9	105.92 (16)
N22—Fe1—N25	86.68 (5)	N4—C11—C10	108.20 (16)
N5—Fe1—N25	107.84 (5)	N4—C11—C13	123.53 (17)
N2—Fe1—N1	72.40 (5)	C10—C11—C13	128.27 (18)
N22—Fe1—N1	86.20 (5)	C21—N21—C25	117.96 (16)
N5—Fe1—N1	157.65 (5)	C21—N21—Fe1	127.44 (12)
N25—Fe1—N1	87.65 (5)	C25—N21—Fe1	114.43 (12)
N2—Fe1—N21	86.01 (5)	N21—C21—C22	123.22 (18)
N22—Fe1—N21	72.10 (5)	C21—C22—C23	118.3 (2)
N5—Fe1—N21	89.10 (5)	C24—C23—C22	119.36 (19)
N25—Fe1—N21	156.60 (5)	C23—C24—C25	118.91 (18)
N1—Fe1—N21	81.38 (5)	N21—C25—C24	122.18 (17)
C5—N1—C1	118.35 (16)	N21—C25—C26	114.32 (15)
C5—N1—Fe1	126.76 (13)	C24—C25—C26	123.49 (16)
C1—N1—Fe1	114.89 (11)	N22—C26—C28	109.37 (17)
N1—C1—C2	121.87 (18)	N22—C26—C25	117.15 (14)
N1—C1—C6	114.42 (15)	C28—C26—C25	133.48 (17)
C2—C1—C6	123.70 (17)	C26—N22—N23	107.61 (13)
C3—C2—C1	118.8 (2)	C26—N22—Fe1	119.67 (11)
C4—C3—C2	119.41 (19)	N23—N22—Fe1	130.40 (11)
C3—C4—C5	118.7 (2)	C27—N23—N22	108.89 (15)
N1—C5—C4	122.9 (2)	C27—N23—B21	129.91 (16)
N2—C6—C8	109.69 (16)	N22—N23—B21	120.69 (13)
N2—C6—C1	116.60 (15)	N23—C27—C28	109.43 (17)
C8—C6—C1	133.68 (17)	C27—C28—C26	104.69 (16)
C6—N2—N3	107.60 (13)	N23—B21—N24	109.44 (15)
C6—N2—Fe1	119.29 (11)	C31—N24—N25	109.38 (13)
N3—N2—Fe1	130.43 (11)	C31—N24—B21	127.18 (15)
C7—N3—N2	108.96 (15)	N25—N24—B21	122.83 (14)
C7—N3—B1	130.21 (16)	C29—N25—N24	106.11 (13)
N2—N3—B1	120.03 (14)	C29—N25—Fe1	125.55 (11)
N3—C7—C8	109.22 (16)	N24—N25—Fe1	124.62 (10)
C7—C8—C6	104.53 (16)	N25—C29—C30	110.40 (15)
N3—B1—N4	109.16 (15)	N25—C29—C32	122.39 (17)
C11—N4—N5	109.56 (14)	C30—C29—C32	127.20 (17)
C11—N4—B1	126.75 (15)	C31—C30—C29	105.85 (16)
N5—N4—B1	122.65 (14)	N24—C31—C30	108.26 (16)
C9—N5—N4	106.14 (13)	N24—C31—C33	123.06 (17)
C9—N5—Fe1	126.18 (11)	C30—C31—C33	128.65 (18)
N4—N5—Fe1	124.57 (10)		

### Hydrogen-bond geometry (Å, º)

**Table:**

*D*—H···*A*	*D*—H	H···*A*	*D*···*A*	*D*—H···*A*
C3—H3···N25^i^	0.95	2.60	3.502 (4)	159

Symmetry code: (i) *x*, *y*−1, *z*.

### Selected bond lengths [Å] and angles [°] for the title compound at 200 K.

**Table:**

Fe(1)-N(2)	2.1222 (13)	Fe(1)-N(25)	2.1866 (14)
Fe(1)-N(22)	2.1264 (13)	Fe(1)-N(1)	2.2972 (14)
Fe(1)-N(5)	2.1782 (14)	Fe(1)-N(21)	2.3255 (15)
Average bond length	2.206		
			
N(2)-Fe(1)-N(5)	86.86 (5)	N(22)-Fe(1)-N(1)	86.20 (5)
N(22)-Fe(1)-N(5)	110.14 (5)	N(25)-Fe(1)-N(1)	87.65 (5)
N(2)-Fe(1)-N(25)	110.34 (5)	N(2)-Fe(1)-N(21)	86.01 (5)
N(22)-Fe(1)-N(25)	86.68 (5)	N(22)-Fe(1)-N(21)	72.10 (5)
N(5)-Fe(1)-N(25)	107.84 (5)	N(5)-Fe(1)-N(21)	89.10 (5)
N(2)-Fe(1)-N(1)	72.40 (5)	N(1)-Fe(1)-N(21)	81.38 (5)
