# Supplementary figures and images for: Crystal structure of bis­{(3,5-di­methyl­pyrazol-1-yl)di­hydro­[3-(pyridin-2-yl)pyrazol-1-yl]­borato}iron(II)
Source: Acta Crystallogr E Crystallogr Commun. 2020 Jul 10;76(Pt 8):1266–70. doi: 10.1107/S2056989020009214 (PMC7405558; doi:10.1107/S2056989020009214)

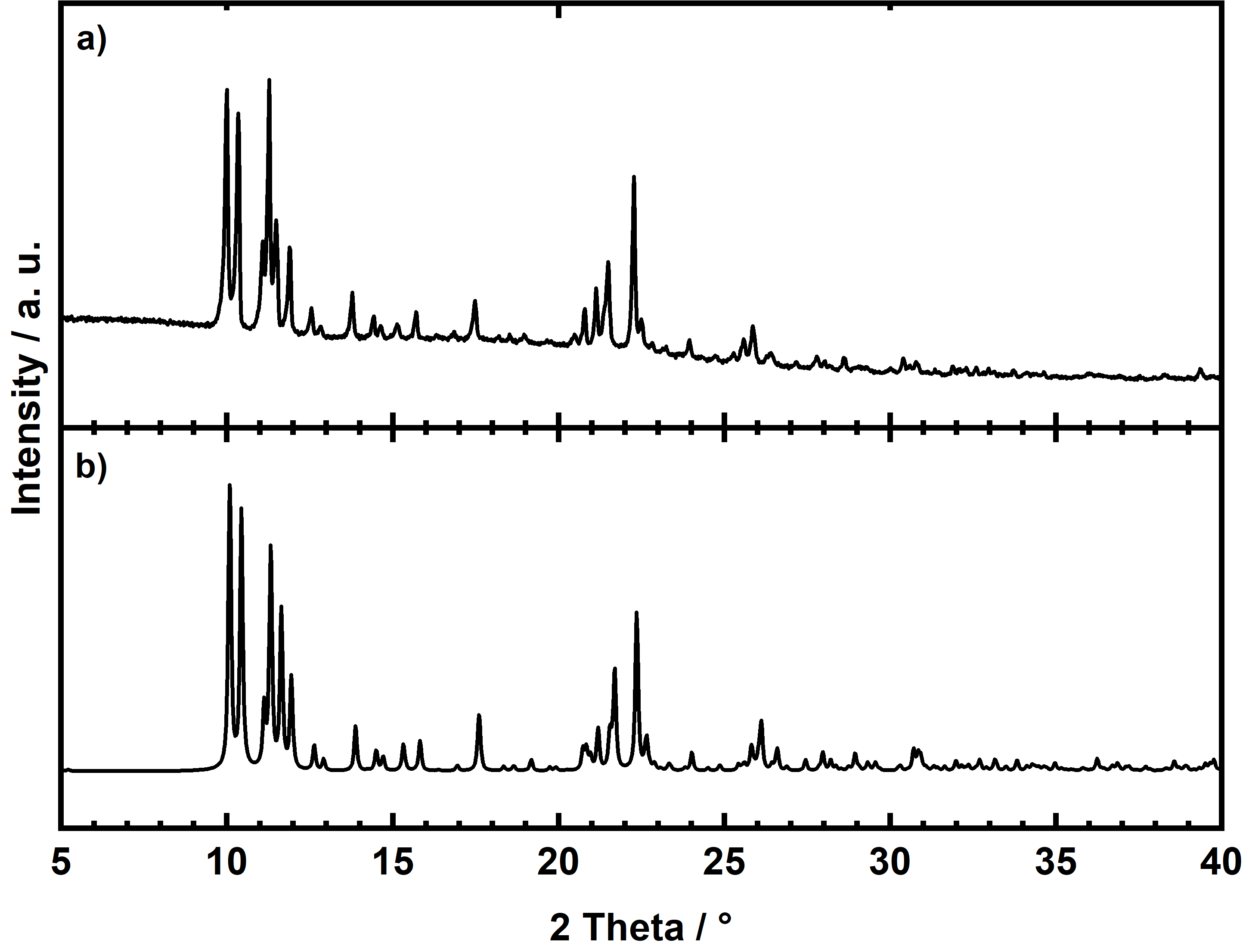

Supplement: Supplementary file 3 [file e-76-01266-sup3.png]

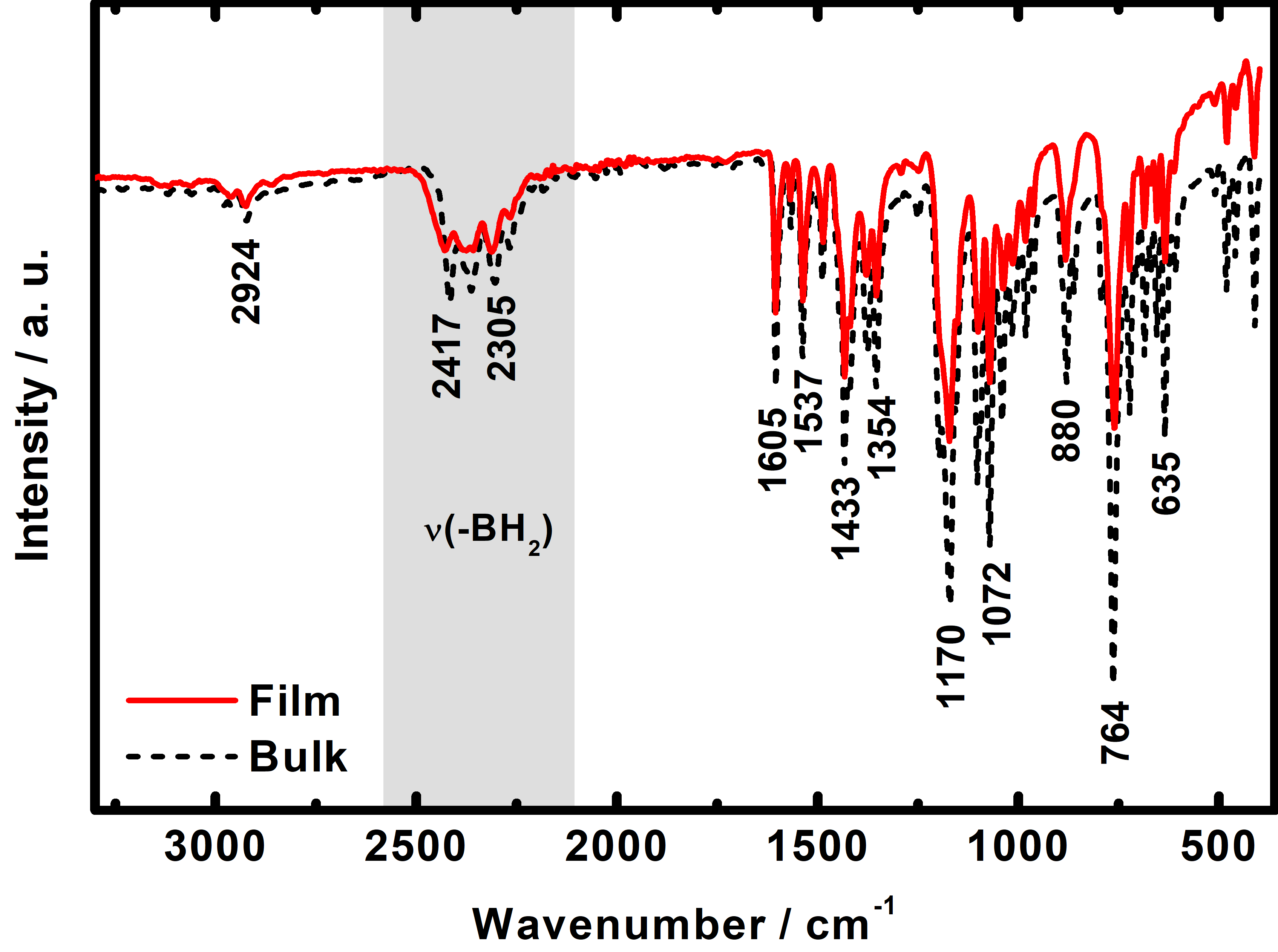

Supplement: Supplementary file 4 [file e-76-01266-sup4.png]

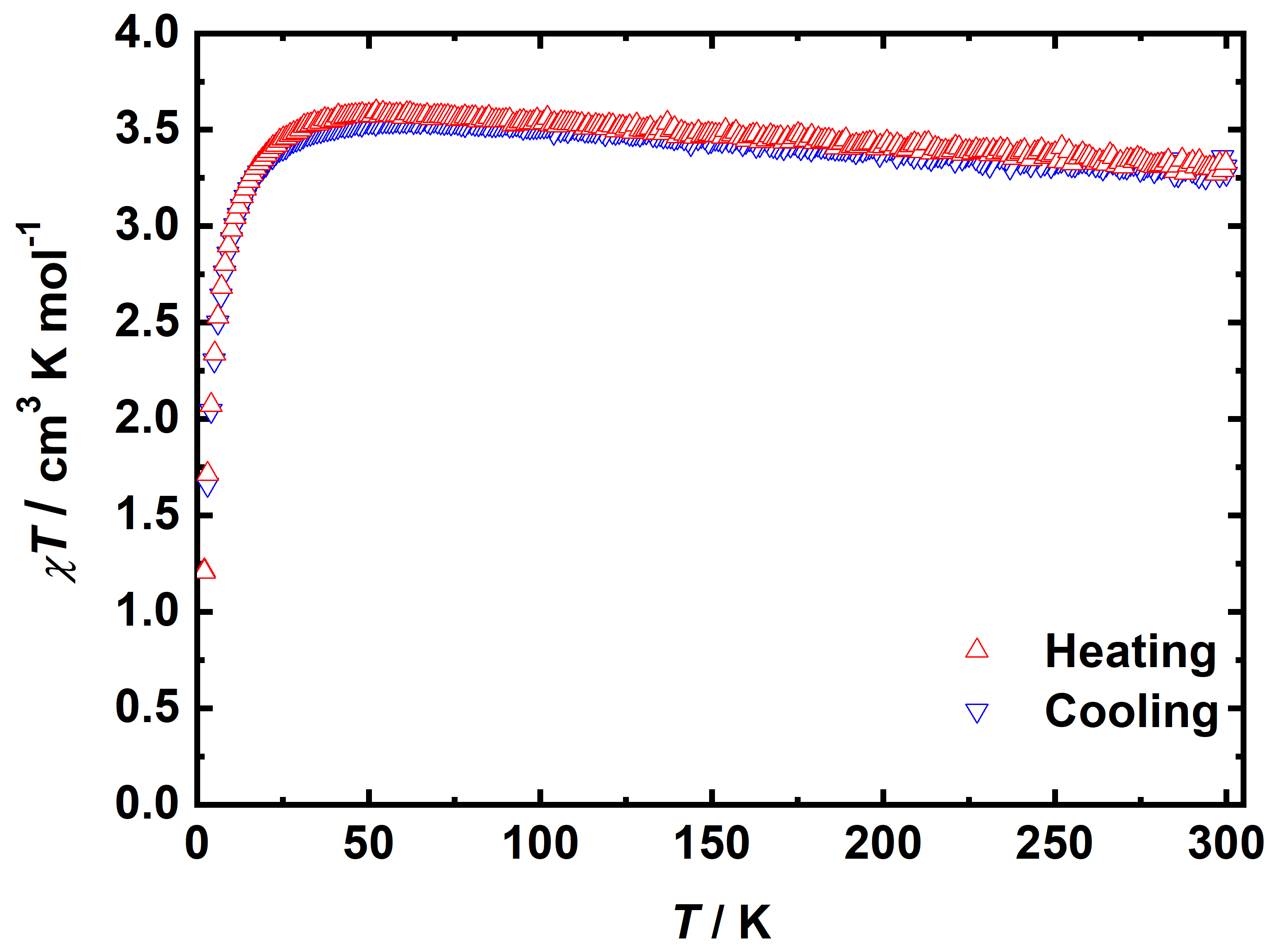

Supplement: Supplementary file 5 [file e-76-01266-sup5.png]
